# Neuroprotective effects of donepezil against cholinergic depletion

**DOI:** 10.1186/alzrt215

**Published:** 2013-10-24

**Authors:** Debora Cutuli, Paola De Bartolo, Paola Caporali, Anna Maria Tartaglione, Diego Oddi, Francesca Romana D’Amato, Annalisa Nobili, Marcello D’Amelio, Laura Petrosini

**Affiliations:** 1 IRCCS Fondazione Santa Lucia, Via del Fosso di Fiorano 64, Rome 00143, Italy; 2 Department of Psychology, University of Rome “Sapienza”, Via dei Marsi 78, Rome 00185, Italy; 3 Cell Biology and Neurobiology Institute, National Research Council, Via del Fosso di Fiorano 64, Rome 00143, Italy; 4 Medical School, Campus Bio-Medico University Molecular Neuroscience Unit, Via Alvaro del Portillo 21, Rome 00128, Italy

## Abstract

**Introduction:**

Intraparenchymal injections of the immunotoxin 192-IgG-saporin into medial septum and nucleus basalis magnocellularis causes a selective depletion of basal forebrain cholinergic neurons. Thus, it represents a valid model to mimic a key component of the cognitive deficits associated with aging and dementia. Here we administered donepezil, a potent acetylcholinesterase inhibitor developed for treating Alzheimer’s disease, 15 days before 192-IgG-saporin injection, and thus we examined donepezil effects on neurodegeneration and cognitive deficits.

**Methods:**

Caspase-3 activity and cognitive performances of lesioned rats pre-treated with donepezil or saline were analyzed and compared to the outcomes obtained in pre-treated sham-lesioned rats.

**Results:**

Cholinergic depletion increased hippocampal and neocortical caspase-3 activity and impaired working memory, spatial discrimination, social novelty preference, and ultrasonic vocalizations, without affecting anxiety levels and fear conditioning. In lesioned animals, donepezil pre-treatment reduced hippocampal and neocortical caspase-3 activity and improved working memory and spatial discrimination performances and partially rescued ultrasonic vocalizations, without preventing social novelty alterations.

**Conclusions:**

Present data indicate that donepezil pre-treatment exerts beneficial effects on behavioral deficits induced by cholinergic depletion, attenuating the concomitant hippocampal and neocortical neurodegeneration.

## Introduction

Experimental and clinical evidence supports the hypothesis that the loss of basal forebrain (BF) cholinergic neurons and the consequent reduction of acetylcholine (ACh) synthesis and release significantly contribute to the cognitive impairment of aging disorders, such as mild cognitive impairment (MCI) and Alzheimer’s disease (AD) [1-3]. Acetylcholinesterase inhibitors (AChE-Is) such as donepezil prevent the hydrolysis of the residual ACh in the brain and represent the best pharmacological tool to attenuate cognitive disturbances in patients with mild to moderate AD [4,5]. AChE-Is are currently used as a symptomatic treatment to improve or at least maintain central cholinergic function [6,7].

To date, besides the research of new drugs able to combat age-related cognitive decline, the protection of neurons from damage and death associated with neurodegenerative disorders is a major challenge in neuroscience. The concept of neuroprotection has found increasing acceptance in neurology during the past decade and includes interventions aimed to slow or even halt the progress of neuronal degeneration. Interestingly, there is growing evidence that, beyond allowing alleviation of cognitive symptoms, AChE-Is produce effective neuroprotection [7]. In fact, it has been shown that AChE-Is protect against glutamate excitotoxicity, neuronal damage and amyloid β (Aβ) neurotoxicity. Furthermore, many studies have shown that they induce upregulation of nicotinic ACh receptors (nAChRs) [8-18]. Importantly, α_4_ and α_7_ nAChRs play a crucial role in AChE-I-mediated neuroprotection, mainly through the involvement of the phosphatidylinositol 3-kinase (PI3K) pathway [8,17]. Unfortunately, few *in vivo* studies have examined AChE-I neuroprotective action [7]. Although a lot of studies have demonstrated the symptomatic effects of donepezil in models of aging and dementia [19-25], a handful of studies have distinguished symptomatic from neuroprotective effects by administering donepezil only *before* (not *during*) behavioral testing [12-14,26,27]. Namely, injecting donepezil before a hypoxic insult has been shown to alleviate hypoxia-induced neurodegeneration and behavioral impairment [12], and, similarly, administrating donepezil before Aβ injection was demonstrated to block lipid peroxidation and learning deficits [13]. In both studies, donepezil neuroprotective effects appeared to be mediated by the activation of the σ_1_ receptor, a protein involved in modulation of intracellular Ca^2+^ mobilization, oxidative stress and neurotransmitter response. Furthermore, donepezil pre-treatment significantly prevented isoflurane-induced cholinergic degeneration and spatial memory impairment in aged mice [27] and attenuated okadaic acid–induced memory impairment, mitochondrial dysfunction and apoptotic cell death [26]. Donepezil pretreatment also prevented streptozotocin (STZ)-induced memory deficits, oxidative stress, mitochondrial dysfunction and caspase-3 activity through the specific involvement of nAChRs [14].

In the light of these studies, it seemed interesting to assess the neuroprotective properties of long-term pre-lesion donepezil treatment in a rat model of BF cholinergic depletion induced by 192-immunoglobulin G (IgG)-saporin (Sap) injection. The resulting permanent and selective Sap-dependent loss of cholinergic BF neurons mimics neuropathological features and cognitive impairments associated with MCI and AD. In fact, Sap selectively causes death of cholinergic cells by inhibiting ribosomal protein synthesis when it is taken up into cells expressing the low-affinity neurotrophin receptor p75 [28]. In the past two decades, the availability of Sap allowed studying the role of BF cholinergic system in several cognitive functions and its implications in aging and dementia [29].

In the present study, we focused on the neuroprotective action of donepezil by investigating the influence of donepezil pre-treatment on cognitive deficits and neuronal impairment induced by intraparenchymal Sap injections into the medial septum (MS) and nucleus basalis magnocellularis (NBM). To achieve this aim, cognitive performance and caspase-3 activity levels of cholinergically depleted rats pre-treated with donepezil or saline were compared with those of pre-treated sham-lesioned animals. Cognitive function was analyzed by means of a wide battery of behavioral tests, including elevated plus maze (EPM), open field with objects (OF), radial arm maze (RAM), sociability and preference for social novelty test (PSNT) and fear conditioning (FC) with ultrasonic vocalization (USV) recording. The low-frequency USVs reflect a negative affective state [30,31] and are positively correlated with the aversiveness of the situation [32]. After behavioral testing, neurodegeneration was analyzed by measuring caspase-3 activity in the hippocampus and neocortex, projection areas of the lesion sites. In fact, caspase-3 is the main effector caspase, whose localized activation can trigger synaptic loss, causing cognitive and behavioral deficits [33], whereas strong activation leads to switching on of the apoptotic cascade. Because the increase in caspase-3 activity has been proposed as an early neurodegenerative event in AD progression [34-36], its quantification might be useful in evaluating the efficacy of neuroprotective pharmacological treatment.

## Methods

### Study animals

Male Wistar rats (about 300 g and 2.5 months of age) kept in standard laboratory conditions (08.00:20.00 light, food and water *ad libitum* and controlled humidity and temperature) were used in the experiments. The animals were maintained according to the guidelines for ethical conduct developed by the European Union directive of 22 September 2010 (2010/63/EU). The study was approved by the local ethics committee of the IRCCS Fondazione Santa Lucia. Rats subjected to behavioral testing were randomly assigned to the following experimental groups: (1) donepezil-treated sham-lesioned (Don-Sham) rats (*n* = 7), which were treated with donepezil and then sham-lesioned; (2) donepezil-treated Sap-lesioned (Don-Sap) rats (*n* = 8), which were treated with donepezil and then Sap-lesioned; (3) saline-treated Sap-lesioned (Sal-Sap) rats (*n* = 8), which were treated with saline (NaCl 0.9%) and then Sap-lesioned; and (4) saline-treated sham-lesioned (Sal-Sham) rats (*n* = 12), which were treated with saline and then sham-lesioned. This group encompassed six intact rats treated with saline and six rats pre-treated with saline and then sham-lesioned. The behavioral performance of the two groups was not statistically different in all the following behavioral parameters (multivariate analysis of variance (group × parameter): group: F_1,10_ = 0.01, *P =* n.s.; parameter: F_37,370_ = 120.79, *P <* 0.000001; and group × parameter: F_37,370_ = 1.25, *P =* n.s.; Additional file 1). These animals were pooled in the Sal-Sham group. At the end of behavioral testing, all rats were killed to verify the lesion by choline acetyltransferase (ChAT) immunohistochemistry on the lesion sites (MS and NBM). Furthermore, an additional three rats per group were prepared to verify ChAT levels in target areas of cholinergic projections (hippocampus and neocortex) and synaptic impairment. In these rats, ChAT densitometry and caspase-3 activity were measured 2.5 weeks after Sap or sham surgery.

### Drug

**Figure 1:**
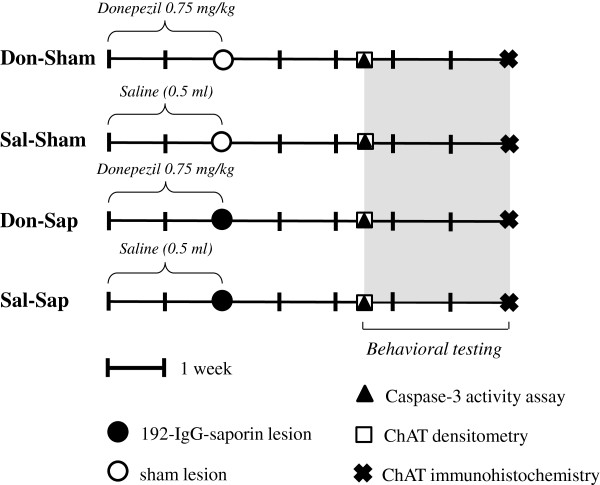
**Experimental design.** Diagram describing the global timing of the experimental design of the four groups. Data derived from pre-treatment (donepezil or saline), lesioned (192-IgG-saporin, Sap, or sham), caspase-3 and choline acetyltransferase analyses, as well asbehavioral testing, are indicated. ChAT, Choline acetyltransferase; Don-Sap, donepezil-treated Sap-lesioned rats; Don-Sham, donepezil-treated sham-lesioned rats; IgG, immunoglobulin G; Sal-Sap, saline-treated Sap-lesioned rats; Sal-Sham, saline-treated sham-lesioned rats.

Donepezil hydrochloride, (*RS*)*-2-*[(*1-benzyl-4-piperidyl*)*methyl*]*-5,6-dimethoxy-2,3-dihydroinden-1-one* (Aricept; Pfizer Inc, New York, NY, USA), was intraperitoneally (i.p.) administered daily at a dosage of 0.75 mg/kg dissolved in 0.5 ml of 0.9% NaCl solution. The same volume of saline without the drug was administered daily to the animals used as controls (Sal-Sham and Sal-Sap). Pre-treatment started 15 days before Sap or sham lesions were created and was stopped after the surgery (Figure 1). The donepezil dosage was chosen on the basis of information from previous *in vivo* studies [12,13].

### Surgery

All rats were anesthetized with a mixture of tiletamine/zolazepam (50 mg/kg Zoletil 100 i.p.; Virbac s.r.l., Milan, Italy) and xylazine (10 mg/kg Rompun i.p.; Bayer s.p.a., Milan, Italy). In the animals to be lesioned, the immunotoxin 192 IgG-Sap (Chemicon International, Harrow, UK) was bilaterally injected through a 10-μl Hamilton syringe in the MS (dosage: 0.5 μg/side 192 IgG-Sap; coordinates: anteroposterior (AP) *=* +0.45 mm (from the bregma); mediolateral (ML) = ±0.6 mm (from the midline); and dorsoventral (DV) = −7.2 mm (from the dura) [36]) and in the NBM (dosage: 0.4 μg/side; coordinates: AP *=* +1 mm (from the bregma); ML = ±2.3 mm (from the midline); and DV = −8 mm (from the dura) [37] with doses and coordinates modified from those used by Frick *et al*. [38]). 192 IgG-Sap diluted in PBS (1 μg:1 μl) was injected at a rate of 0.1 μl/min. At the end of administration, the needle was left *in situ* for five minutes. In the remaining rats, only PBS solution was injected.

### Behavioral testing

As shown in Figure 1, 2.5 weeks after the surgery (time required to reach a stable and permanent loss of cholinergic neurons using Sap [28]), all rats were subjected to the following tests: EPM, to assess anxiety levels; OF, to evaluate the ability to develop spatial and discriminative competencies; RAM, to analyze spatial working memory; sociability test, to analyze social motivation; PSNT, to evaluate social novelty discrimination; and contextual and tone FC with USV recordings, to analyze acquisition of aversive learning and emotional competencies. A pseudo-random order of test administration was used with FC always being the last test, given its high stress levels.

### Elevated plus maze

The maze raised 90 cm above the ground was formed by a wooden structure in the shape of a cross with four 50 cm × 10 cm arms. The north and south arms were open, and the east and west arms were enclosed by walls 36 cm high. The parameters taken into account were frequency of entries into the open and closed arms, total time spent in the open and closed arms and number of defecations [20].

The parameters taken into account were: frequency of entries in the open and closed arms; total time spent in the open and closed arms; number of defecations [20].

### Open field with objects

The apparatus consisted of a circular arena (diameter 140 cm) delimited by a 30-cm-high wall. During session 1 (S1), each rat was allowed to move freely in the empty open field and the baseline activity level was measured. During S2 to S4 (habituation phase), four objects were placed in a square arrangement in the middle annulus of the arena and a fifth one was placed in the central area. In S5 and S6 (spatial change), the spatial configuration was changed by moving objects 2 and 5 so that the initial square arrangement was changed to a polygon-shaped configuration without any central object. During S7 (novelty), the configuration was modified by substituting one object with another new one. Sessions lasted six minutes, and intersession intervals were three minutes long. All testing was recorded by a video camera whose signal was relayed to a monitor and to an image analyzer (EthoVision, Noldus Information Technology, Wageningen, The Netherlands). The parameters taken into account were total and peripheral distances traveled in S1, time spent contacting the objects, frequency of rearing, motionless time and number of defecations [20,39].

### Radial arm maze

The apparatus consisted of a central platform (30-cm diameter) from which eight arms (12.5 cm wide × 60 cm long) radiated like the spokes of a wheel. A food well (5-cm diameter, 2-cm depth) was located at the end of each arm [20]. A 40-W red light bulb provided the only source of illumination in the testing room. Testing was performed between 09:00 and 17:00 hours. After a habituation phase, all rats (whose food was restricted to decrease their weight by approximately 15%) were subjected to the RAM full-baited procedure in which all arms were baited with a piece of Purina chow (Purina Mills, Gray Summit, MO, USA) with the goal of having the rats collect the eight rewards in a maximum of 16 entries. The animals were subjected to two trials daily for five consecutive days. The intersession interval was four hours. The parameters taken into account were percentage of total errors (number of revisited arms divided by total number of visits × 100), mean spatial span (longest sequence of correctly visited arms in each session) and perseverations (sum of consecutive entries in the same arm or in a fixed sequence of arms).

### Sociability and preference for social novelty test

The apparatus consisted of a white rectangular three-chamber wooden box (150 × 40 × 40 cm). The central chamber was 30 cm long, and the two lateral chambers were 60 cm long. The three chambers were divided by two transparent Plexiglas walls with an open middle door (height 10 cm, width 8 cm), which allowed free access to each lateral chamber. Each lateral chamber contained a small plastic cage (18-cm diameter) with meshlike holes in which stranger rats were confined for social interaction.

The test comprised 3 sessions: Habituation, Sociability and PSNT. During the habituation, each rat was allowed to freely move in the apparatus for 10 min. During Sociability, an unfamiliar juvenile (35 to 45 days old) male conspecific (Stranger 1) was placed inside the small cage in one of the lateral chambers (randomly selected and counterbalanced for each group). The experimental rat was placed in the apparatus and it was allowed to freely explore the three chambers and contact the small cages for ten minutes.

During PSNT, another unfamiliar rat (stranger 2) was placed inside the plastic cage of the opposite lateral chamber that had been empty during the Sociability session. The experimental rat was allowed to move freely and contact the plastic cages housing the strangers for ten minutes. Inter-session intervals lasted three minutes. Rats’ behavior was recorded by a video camera mounted on the ceiling. The resulting video signal was relayed to a monitor and to an EthoVision image analyzer. The parameters analyzed in each lateral chamber were frequency of entries, total duration (in seconds) and total distance traveled (in centimeters).

### Context and tone fear conditioning

The apparatus consisted of a 21 × 21 × 49 cm conditioning chamber (model 7532; Ugo Basile, Comerio, Italy). Chamber walls were made of gray Plexiglas, and the ceiling was made of transparent Plexiglas to allow video recording. The grid floor (steel pieces spaced by 1.5 cm) was connected to a shock generator scrambler (conditioner 7531; Ugo Basile).

The FC test encompassed three sessions: training, context and tone. During training session, following a 120 seconds of acclimation to the conditioning apparatus (baseline), three trials consisting of the 30-second tone exposure (2 kHz, 90 dB) were carried out. The last 2 seconds of each tone were paired with a 1-mA foot shock. Tone- and shock-free 60-second inter-trial intervals were used.

After 24 hours, rats were placed for 240 seconds in the training chamber (context test). After 4 hours, the rats were submitted to a tone test in a white Plexiglas box (21 × 18 × 45 cm) with black stripes applied on the walls. After 120 seconds of acclimation, a 120-second tone identical to that used in the training session was sounded without any shock (tone test).

During training, context and tone tests, the rats’ behavior was recorded by a video camera mounted on the ceiling. The resulting video signal was relayed to a monitor and to an EthoVision image analyzer. In addition, 22-kHz USVs were recorded [38,40].

Behavioral parameters taken into account were frequency and duration of freezing (behavioral immobility, except for respiration movements) and number of defecations.

USVs were collected via an ultrasound microphone (UltraSoundGate CM16; Avisoft Bioacoustics, Berlin, Germany) placed through a hole in the middle of the test chamber roof approximately 21 cm above the shock floor. The microphone was sensitive to 15 to 180 kHz frequencies with a flat frequency response (±6 dB) between 25 and 140 kHz. It was connected by an UltraSoundGate USB audio device to a personal computer, which recorded data at 250,000 Hz in 16-bit format and stored as .wav files for subsequent analysis. Sound files were transferred to SASLab Pro (version 5.2; Avisoft Bioacoustics) for sonographic analysis and a fast Fourier transform (FFT) was performed (512 FFT length, 100% frame, Hamming window and 75% time window overlap). USV parameters taken into account were number of calls emitted, peak amplitude and frequency, frequency modulation and call duration.

### Histological analyses

At the end of behavioral testing, the animals were deeply anesthetized and transcardially perfused with saline, followed by 4% paraformaldehyde and 0.1% glutaraldehyde in PBS (4°C, pH 7.5). Brains were removed and cryoprotected in 30% buffered sucrose and cut on a freezing microtome. The anterior part of the brain was cut into coronal sections of 40 μm and stored for ChAT immunohistochemistry.

#### Choline acetyltransferase immunohistochemical staining

Sections (40 μm) immunostained for ChAT were preincubated in PBS at 4°C, then in 0.4% Triton X-100 in PBS and finally in 0.1% Triton X-100 plus 1% bovine serum albumin (Sigma-Aldrich, St Louis, MO, USA) plus normal goat serum (NGS; Vector Laboratories, Burlingame, CA, USA) in PBS. Sections were incubated for 16 hours at 4°C with 0.1% Triton X-100 and NGS in PBS with the primary antibody for ChAT diluted 1:1,000 (Chemicon International). Subsequently, sections were incubated with biotinylated secondary antibody (goat anti-rabbit IgG-biotin conjugate) and 3% NGS (Kit Elite PK-6101; Vector Laboratories) in PBS for 10 minutes at room temperature. Staining was visualized with 0.05% diaminobenzidine and ammonium nickel(II) sulfate (Sigma-Aldrich Chemie, Steinheim, Germany) after incubation with avidin and biotinylated peroxidase (Kit Elite PK-6101). The sections were then rinsed in PBS. Stained sections were mounted on slides, dehydrated and coverslipped. To exclude artefacts, in each case some random sections were processed as previously described. The only difference was the absence of the primary antibody.

### Biochemical analyses

#### Total homogenate preparation from hippocampal and neocortical tissues

After the animals were decapitated, hippocampal and neocortical tissues were dissected and homogenized in lysis buffer (320 mM sucrose, 50 mM NaCl, 50 mM Tris-HCl (pH 7.5), 1% Triton X-100, 0.5 mM sodium orthovanadate, 5 mM β-glycerophosphate, proteases inhibitors), then incubated on ice for 30 minutes and centrifuged at 13,000 *g* for 10 minutes. The total protein content of the resulting supernatant was determined by the Bradford assay method.

#### Immunoblot analysis and antibodies

Proteins were subjected to SDS-PAGE and electroblotted onto a polyvinylidene fluoride membrane. Immunoblot analysis was performed using a chemiluminescence detection kit. The relative levels of immunoreactivity were determined by densitometry using ImageQuant 5.0 software. Antibodies to anti-ChAT were purchased from Chemicon International (AB143), and anti-actin clone EP184E rabbit monoclonal antibody was obtained from EMD Millipore (04-1040; Billerica, MA, USA).

#### Fluorometric assay of caspase-3 activity

Total hippocampal and neocortical tissue was homogenized in lysis assay buffer (100 mM 2-[4-(2-hydroxyethyl)piperazin-1-yl]ethanesulfonic acid (pH 7.4), 0.1% 3-[(3-cholamidopropyl)dimethylammonio]-1-propanesulfonate (wt/vol), 1 mM ethylenediaminetetraacetic acid, 10 mM dithiothreitol, 1 mM phenylmethylsulfonyl fluoride) and lysed by freezing in liquid N_2_ and thawing at 37°C three times. After centrifugation at 11,500 *g* for 5 minutes, the protein concentration of resulting supernatant was determined and the same amount of protein was incubated at 37°C in lysis assay buffer containing 50 μM caspase-3 substrate II, fluorogenic (Ac-DEVD-AMC; Calbiochem, San Diego, CA, USA). The fluorescence was measured with 380-nm excitation wavelength and 460-nm emission wavelength.

### Statistical analysis

All data were tested for normality (Shapiro-Wilk test) and homoscedasticity (Levene’s test). Behavioral data were analyzed using two-way analysis of variance (ANOVA; F-statistic) (with drug and lesion as between-animal factors) or a mixed model of three-way ANOVA (with drug and lesion as between-animal factors and arm/session/object/day/chamber as within-animal factors). *Post hoc* comparisons were performed by means of Tukey’s honestly significant difference test. When parametric assumptions were not fully met, data transformations (angular transformation for percentages) or nonparametric ANOVAs (Kruskal-Wallis test (H-statistic) and Mann-Whitney *U* test; Z-statistic) were used. Biochemical data of ChAT levels were analyzed by Student’s *t*-test, and data regarding caspase-3 activity were analyzed using the Bonferroni multiple comparisons test. Differences were considered significant at the *P* ≤ 0.05 level.

## Results

### Lesion verification by choline acetyltransferase immunohistochemical staining

**Figure 2:**
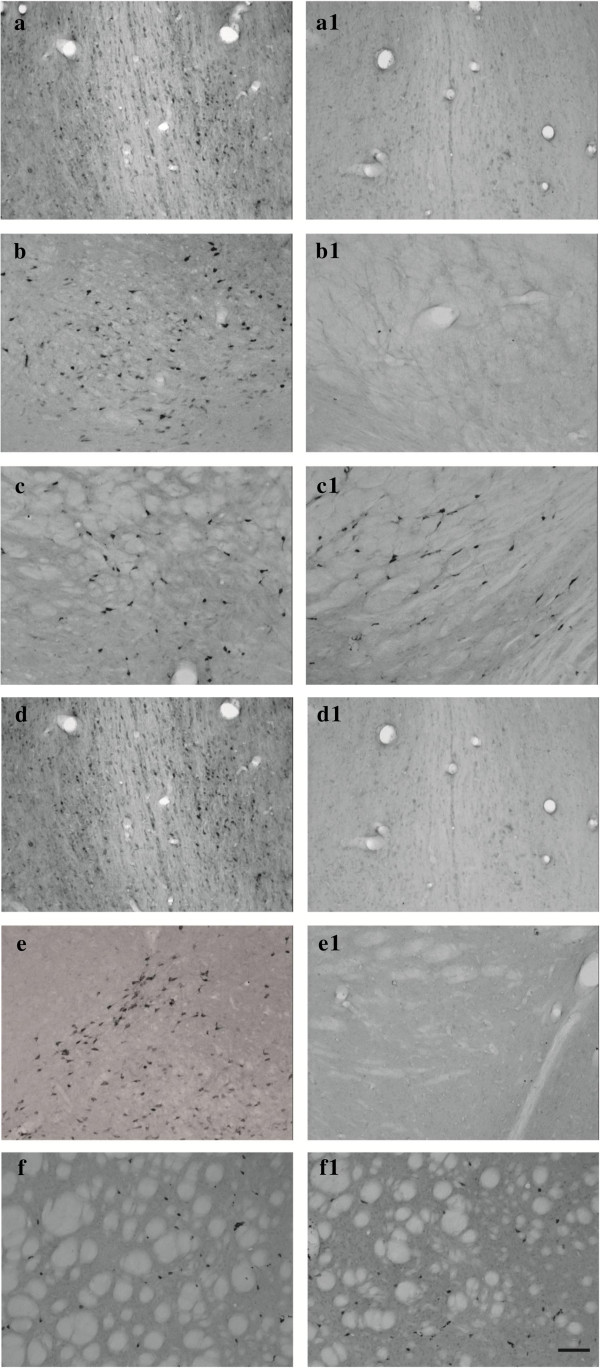
**Choline acetyltransferase immunohistochemical staining.** Representative photomicrographs of intraparenchimal Sap injection effects. Images show brain sections of rats that were saline-treated, sham-lesioned **(a, b and c)**; saline-treated, Sap-lesioned **(a1, b1 and c1)**; donepezil-treated, sham-lesioned **(d, e and f)**; and donepezil-treated, Sap-lesioned **(d1, e1 and f1)**. Coronal sections at the level of medial septum **(a, a1, d and d1)**, nucleus basalis magnocellularis **(b, b1, e and e1)** and striatal **(c, c1, f and f1)** regions with choline acetyltransferase immunoreactive neurons are shown. Note the substantial absence of cholinergic neurons in both regions of the lesioned animal **(a1, b1, d1 and e1)** and the substantial preservation of striatal cholinergic interneurons **(c1 and f1)**. Lens objective: 10×. Scale bars: 50 μm.

The presence of ChAT immunoreactive (ChAT-IR) neurons in the BF projection areas was assessed by inspection (Figures 2(a), 2(a1), 2(b), 2(b1), 2(d), 2(d1), 2(e) and 2(e1)). Brain sections were visualized with the light microscope-interfaced software Neurolucida (MBF Bioscience, Williston, VT, USA). Using a 10× lens objective, ChAT-IR neurons were assessed in the two main regions of the BF: the MS, taking into account five 40-μm sections between 1.20 and 0.20 mm anterior to bregma, and the NBM, taking into account eight 40-μm sections between 0.80 and 2.30 mm posterior to the bregma [37]. Additional visual inspection was carried out to exclude eventual degeneration of striatal cholinergic interneurons after i.p. Sap injections in the NBM (Figures 2(c), 2(c1), 2(f) and 2(f1)).

### Lesion verification by choline acetyltransferase immunoblot analysis

**Figure 3:**
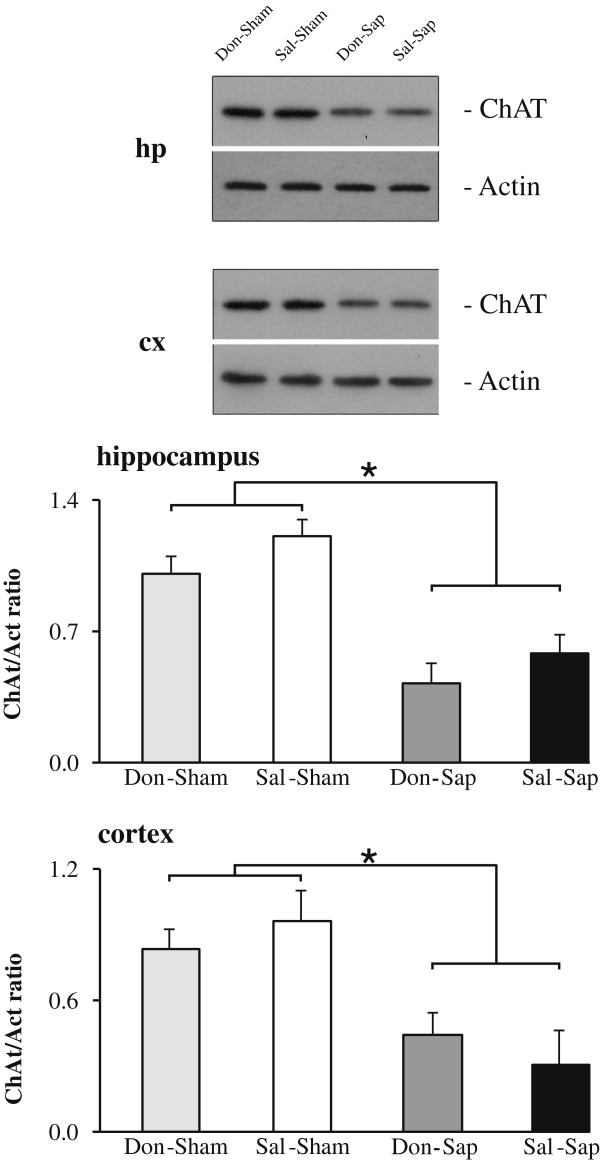
**Choline acetyltransferase immunoblot analysis.** Representative choline acetyltransferase (ChAT) immunoblots of hippocampal (hp) and neocortical (cx) total protein extracts and densitometric quantification of changes in gray values expressed as mean ± SD. The relative fold change in the levels of ChAT protein was normalized with respect to the level of actin, which was used as a loading control. Statistical differences were observed between the sham and 192-IgG-saporin (Sap)-lesioned animals (*n* = 3/group). *P < 0.05 by *t*-test. Don-Sap, donepezil-treated Sap-lesioned rats; Don-Sham, donepezil-treated sham-lesioned rats; Sal-Sap, saline-treated Sap-lesioned rats; Sal-Sham, saline-treated sham-lesioned rats.

Intraparenchymal Sap injections in the NBM and MS induced an extensive loss of ChAT-IR in the synaptic boutons of the neocortex and hippocampus, as demonstrated by a strong reduction in ChAT expression (Figure 3). A comparable reduction of ChAT expression was detected in the hippocampi and neocortices from both lesioned groups (Don-Sap and Sal-Sap). Conversely, ChAT expression was not significantly different in the sham-lesioned groups (Sal-Sham and Don-Sham).

### Hippocampal and neocortical caspase-3 activity

**Figure 4:**
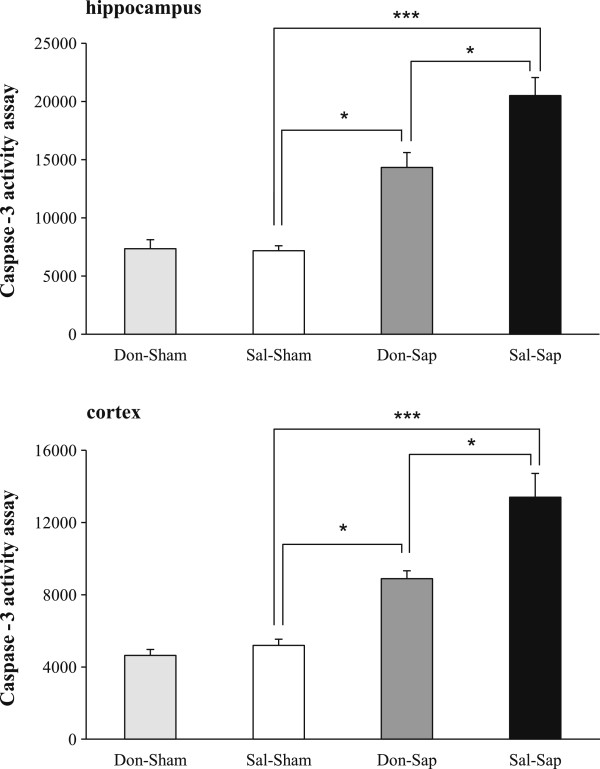
**Hippocampal and neocortical caspase-3 activity.** Caspase-3 activity was revealed by a fluorometric assay in total hippocampal and neocortical homogenates from sham and lesioned rats (*n* = 3/group). The fluorometric data are expressed as mean ± *SEM*. *P < 0.05, ***P ≤ 0.001 (Bonferroni multiple comparisons test). Don-Sap, donepezil-treated Sap-lesioned rats; Don-Sham, donepezil-treated sham-lesioned rats; Sal-Sap, saline-treated Sap-lesioned rats; Sal-Sham, saline-treated sham-lesioned rats.

A significant increase in caspase-3 activity was evident in the Sal-Sap group; however, a partial but significant rescue was found in the Don-Sap group in both hippocampal and neocortical extracts. Both sham-lesioned groups (Don-Sham and Sal-Sham) exhibited similar levels of caspase-3 activity in both hippocampal and neocortical extracts (Figure 4).

### Elevated plus maze

The animals of all groups entered more frequently (F_1,31_ = 47.94; *P <* 0.000001) and spent more time (F_1,31_ = 200.66, *P <* 0.000001) in the closed arms than in the open arms. No difference was detected in the total number of defecations. Thus, neither drug nor lesion affected anxiety-related behavior in the EPM (Additional file 2).

### Open field with objects

Two-way ANOVA on total or peripheral distances traveled in S1 did not reveal any significant effect of drug or lesion. A three-way ANOVA (drug × lesion × session) on motionless time failed to reveal any significant effect of drug or lesion. Session effect (F_6,186_ = 5.01, *P <* 0.0001) was significant, because motionless time increased throughout the task in all groups. Lesion × session interaction was also significant (F_6,186_ = 2.42, *P =* 0.03). *Post hoc* comparisons revealed that both lesioned groups (Don-Sap and Sal-Sap) tended to exhibit motionless time lower than that of sham animals (Don-Sham and Sal-Sham) in the final sessions of the test (S6: *P =* 0.02; S7: *P =* 0.07). No other significant interactions were found. As sessions went by, rearing (F_6,186_ = 28.52, *P =* 0.000001) and defecation (F_6,186_ = 14.88, *P =* 0.000001) decreased without any significant differences among groups. For both parameters, no significant drug and lesion effects were observed.

In the presence of the objects, all animals showed habituation, as revealed by the significant session effect (F_2,62_ = 42.26, *P <* 0.000001) of three-way ANOVA (drug × lesion × session) on contact times. Drug and lesion effects and their interaction were not significant.

**Figure 5:**
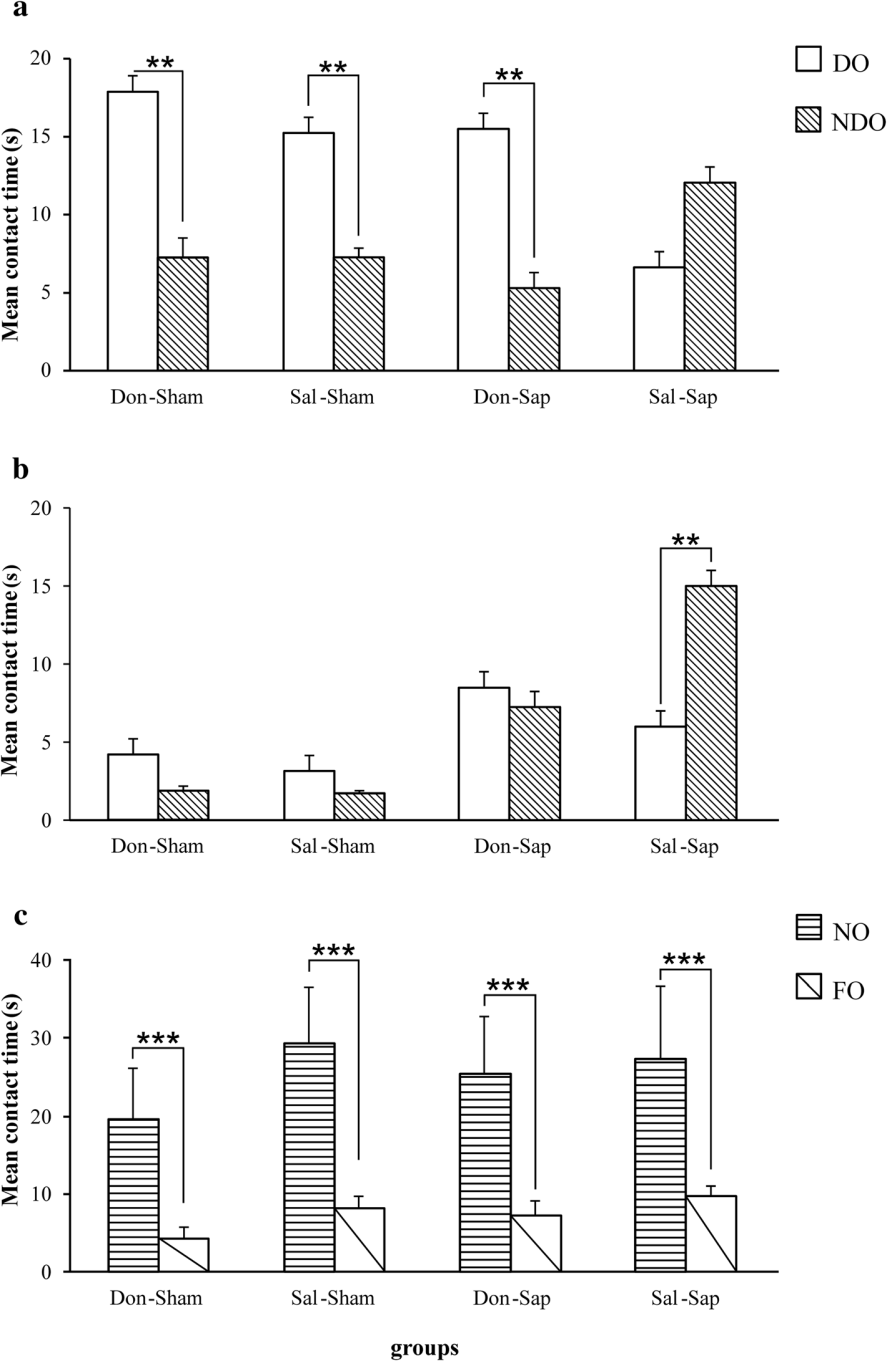
**Open field with objects.** Effects of donepezil pre-treatment and 192-IgG-saporin (Sap) lesions on open field with objects contact time with objects during spatial change in session 5 (S5) **(a)**, S6 **(b)** and novelty (S7) **(c)**. Cholinergic depletion per se (Sal-Sap group) significantly affected spatial discrimination abilities in S5 and S6, and donepezil pre-treatment (Don-Sap group) prevented spatial memory deficits. In S7, all rats detected the presence of the novel object. Behavioral data are expressed as means ± *SEM,* and asterisks indicate *post hoc* comparisons between groups. DO, Displaced object; Don-Sap, donepezil-treated Sap-lesioned rats; Don-Sham, donepezil-treated sham-lesioned rats; FO, familiar object; NDO, non-displaced objects; NO, novel object; Sal-Sap, saline-treated Sap-lesioned rats; Sal-Sham, saline-treated sham-lesioned rats. **P < 0.01, ***P < 0.001.

A three-way ANOVA (drug × lesion × object) on S5 contact time revealed a significant object effect (F_1,31_ = 23.28, *P* < 0.0001), as well as significant interactions of drug × object (F_1,31_ = 14.22, *P* < 0.001), lesion × object (F_1,31_ = 8.11, *P* < 0.01) and drug × lesion × object (F_1,31_ = 7.15, *P* = 0.01). *Post hoc* comparisons performed on the second-order interaction revealed that, in S5, both sham groups (Don-Sham and Sal-Sham), as well as the Don-Sap group, recognized the spatial change (*P* < 0.01), whereas the Sal-Sap rats failed to detect the new spatial configuration (Figure 5(a)). Drug and lesion effects and their interaction were not significant.

A three-way ANOVA (drug × lesion × object) on S6 contact time revealed a significant lesion effect (F_1,31_ = 24.64, *P <* 0.0001), as well as significant interactions of drug × object (F_1,31_ = 13.18, *P =* 0.001), lesion × object (F_1,31_ = 14.05, *P =* 0.001) and drug × lesion × object (F_1,31_ = 9.26, *P =* 0.005). *Post hoc* comparisons on the second-order interaction revealed that, though Don-Sham, Sal-Sham and Don-Sap groups did not show any preference towards displaced objects, Sal-Sap rats continued to explore nondisplaced objects longer (*P* < 0.001) (Figure 5(b)). Drug and object effects and drug × lesion interactions were not significant.

A three-way ANOVA (drug × lesion × object) on S7 contact time indicated that all animals recognized novelty, as revealed by the significant object effect (F_1,31_ = 25.31, *P <* 0.0001) (Figure 5(c)). Drug and object effects and their interactions were not significant.

A three-way ANOVA (drug × lesion × object) on S7 contact time indicated that all animals recognized novelty, as revealed by the significant object effect (F_1,31_ = 25.31, *p <* 0.0001) (Figure 5c). Drug and object effects, and their interactions were not significant.

### Radial arm maze

**Figure 6:**
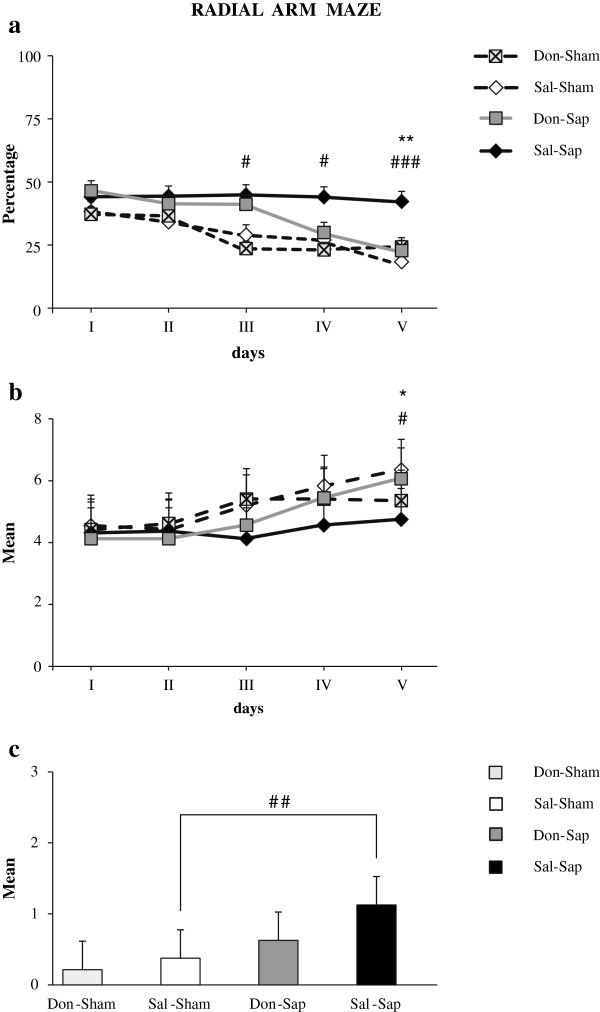
**Radial arm maze.** Effects of donepezil pre-treatment and 192-IgG-saporin (Sap) lesions on radial arm maze total errors **(a)**, spatial spans **(b)** and perseverations **(c)**. Donepezil pre-treatment reduced the number of errors and increased spatial span in cholinergically depleted rats. Sal-Sham *vs*. Sal-Sap: #*p* < 0.05; ##*p* < 0.01; ###*p* < 0.001; Don-Sap *vs*. Sal-Sap: *p = 0.05, **p = 0.01. Don-Sap, donepezil-treated Sap-lesioned rats; Don-Sham, donepezil-treated sham-lesioned rats; Sal-Sap, saline-treated Sap-lesioned rats; Sal-Sham, saline-treated sham-lesioned rats.

As the task went by, all animals but the Sal-Sap rats learned to correctly visit the baited arms. A three-way ANOVA (drug × lesion × day) on total errors revealed significant effects of lesion (F_1,31_ = 16.92, *P <* 0.001) and day (F_4,124_ = 11.26, *P <* 0.000001), as well as a significant second-order interaction (F_4,124_ = 2.75, *P <* 0.05). *Post hoc* comparisons of the interactions revealed that Sal-Sham, Don-Sham and Don-Sap performance was not significantly different. Conversely, Sal-Sap rats made significantly more errors than Sal-Sham and Don-Sap animals, as reported in Figure 6(a). No significant drug effect or first-order interactions were found.

A three-way ANOVA (drug × lesion × day) of spatial span revealed significant day effect (F_4,124_ = 12.19, *P <* 0.000001) and second-order interaction (F_4,124_ = 3.01, *P <* 0.05). *Post hoc* comparisons indicated that Sal-Sap rats exhibited a shorter spatial span than the animals in the Sal-Sham and Don-Sap groups (Figure 6(b)). No significant drug and lesion effects or first-order interactions were found.

The Kruskal-Wallis test demonstrated a significant difference among the four experimental groups (H = 9.45, *P* = 0.02). In fact, a marked presence of perseverations was evident in Sal-Sap animals when compared to Sal-Sham rats (Z = −2.75, *P =* 0.007 by Mann-Whitney *U* test). Conversely, no difference in perseverations was found between the Sal-Sham, Don-Sham and Don-Sap groups. Furthermore, the Don-Sap group tended to perseverate less than the Sal-Sap animals (*P* = 0.08) (Figure 6(c)), indicating a trend toward reducing perseverative behavior in the presence of lesions with donepezil pre-treatment.

### Sociability and preference for social novelty test

#### Habituation

Three-way ANOVA (drug × lesion × chamber) on all parameters during the habituation session allowed us to exclude any side preference, which could have constituted a bias for the following test sessions (Table 1).

**Table 1:** **Statistical comparison of behavioral responses in the habituation, sociability and preference for social novelty test**^**a**^

	**Drug**		**Lesion**		**Chamber**		**Drug × lesion**		**Drug × chamber**		**Lesion × chamber**		**Drug × lesion × chamber**	
**Parameters**	**F (*df *** **= 1, 31)**	***p***	**F (*df *** **= 1, 31)**	***p***	**F (*df *** **= 1, 31)**	***P***	**F (*df *** **= 1, 31)**	***P***	**F (*df *** **= 1, 31)**	***P***	**F (*df *** **= 1, 31)**	***P***	**F (*df *** **= 1, 31)**	***P***
**Habituation**														
Frequency of entries	0.01	n.s.	1.54	n.s.	3.11	n.s.	4.01	n.s.	3.01	n.s.	0.81	n.s.	0.89	n.s.
Total duration	3.62	n.s.	0.30	n.s.	0.38	n.s.	2.68	n.s.	0.01	n.s.	3.61	n.s.	2.92	n.s.
Total distance	1.90	n.s.	1.31	n.s.	0.25	n.s.	0.06	n.s.	0.15	n.s.	3.84	n.s.	3.67	n.s.
**Sociability**														
Frequency of entries	0.01	n.s.	0.21	n.s.	2.95	n.s.	0.47	n.s.	0.01	n.s.	1.06	n.s.	0.47	n.s.
Total duration	0.04	n.s.	0.66	n.s.	57.87	<0.000001	0.04	n.s.	0.19	n.s.	0.02	n.s.	2.47	n.s.
Total distance	0.01	n.s.	0.18	n.s.	64.05	<0.000001	1.71	n.s.	0.43	n.s.	0.05	n.s.	0.91	n.s.
**Preference for social novelty test**														
Frequency of entries	0.28	n.s.	0.01	n.s.	9.13	<0.01	2.41	n.s.	0.11	n.s.	0.18	n.s.	0.12	n.s.
Total duration	1.42	n.s.	0.20	n.s.	2.84	n.s.	0.76	n.s.	0.34	n.s.	4.21	<0.05	0.13	n.s.
Total distance	1.47	n.s.	2.18	n.s.	7.17	0.01	1.25	n.s.	0.27	n.s.	4.48	<0.05	0.48	n.s.

^**a**^All statistical comparisons were carried out by three-way analysis of variance.

#### Sociability

**Figure 7:**
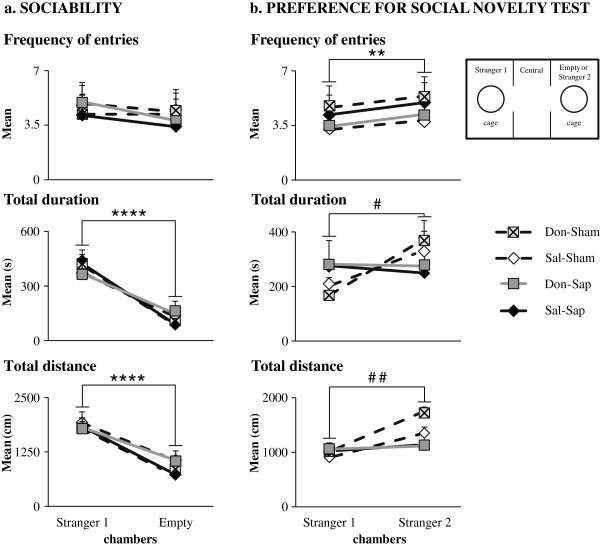
**Sociability and preference for social novelty test.** Effects of donepezil pre-treatment and 192-IgG-saporin (Sap) lesions on sociability **(a)** and preference for social novelty test (PSNT) **(b)**. Although no differences among the four groups were found in the sociability test, both groups of cholinergically depleted animals (Don-Sap and Sal-Sap) failed to exhibit an overt social novelty during PSNT. Asterisks indicate comparisons between stranger 1 and empty/stranger 2 chamber behaviors in the four groups. **P < 0.01, ****P < 0.0001. Pound symbols indicate comparisons between stranger 1 chamber and stranger 2 chamber behaviors in the sham groups (Don-Sham and Sal-Sham). #*P* < 0.05, ##*P* = 0.01. Don-Sap, donepezil-treated Sap-lesioned rats; Don-Sham, donepezil-treated sham-lesioned rats; Sal-Sap, saline-treated Sap-lesioned rats; Sal-Sham, saline-treated sham-lesioned rats.

During the sociability session, all rats showed a marked preference for the stranger 1 chamber (Figure 7(a)) regarding duration and distance (*P* < 0.000001), but not frequency of entries (Table 1).

#### Preference for social novelty test

Don-Sham and Sal-Sham rats showed the predicted preference for the stranger 2 chamber (duration: *P* = 0.04; distance: *P* = 0.01) (Figure 7(b) and Table 1). Conversely, Don-Sap and Sal-Sap rats, although they entered the stranger 2 chamber more frequently (*P* < 0.01), did not exhibit a clear social novelty recognition, as indicated by the time spent and distance traveled in the stranger 2 chamber (Figure 7(b) and Table 1).

### Context and tone fear conditioning

#### Training

**Figure 8:**
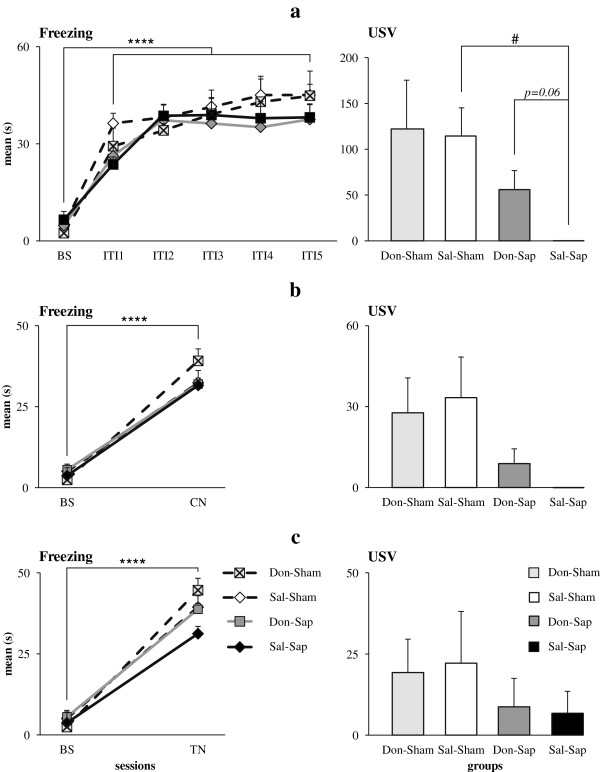
**Fear conditioning.** Effects of donepezil pre-treatment and 192-IgG-saporin (Sap) lesions on percentage of freezing and total number of 22-kHz fear-associated ultrasonic vocalizations (USVs) during fear conditioning (FC) training **(a)**, context (CN) test **(b)** and tone (TN) test **(c)**. All animals acquired a clear freezing response during FC training and showed mnesic retention of the conditioned freezing response during CN and TN tests. USVs were nearly totally suppressed in saline-treated Sap-lesioned rats, and donepezil pre-treatment tended to increase USV production in donepezil-treated Sap-lesioned rats during FC training. BS, Baseline; Don-Sham, donepezil-treated sham-lesioned rats; ITI, Inter-trial interval; Sal-Sham, Saline-treated sham-lesioned rats. All groups: ****P < 0.00001; Sal-Sham *vs.* Sal-Sap: #*P* < 0.05.

In all animals, postshock freezing significantly increased in duration (F_5,155_ = 51.90, *P <* 0.000001) and frequency (F_5,155_ = 22.77, *P <* 0.000001) in comparison to baseline over the course of shock presentation. Neither drug nor lesion affected duration or frequency of freezing (Figure 8(a)). Also, no difference was detected regarding the number of defecations. As for USVs, the Kruskal-Wallis test demonstrated a significant difference among groups (H = 8.21, *P* = 0.04). In fact, Sal-Sap rats vocalized significantly less often than Sal-Sham rats (Z = 2.53, *P =* 0.02 by Mann-Whitney *U* test), whereas no significant differences were found among the remaining groups (Figure 8(a)). Furthermore, Don-Sap rats tended to emit a higher number of USVs than Sal-Sap animals (Z =1.85, *P =* 0.06 by Mann-Whitney *U* test). These findings indicated a trend toward donepezil rescue of USV production in the presence of cholinergic lesion. Other USV parameters are reported in Additional file 3.

#### Context test

During context testing, all animals exhibited a freezing significantly increased in duration (F_1,31_ = 350.98, *P <* 0.000001) and frequency (F_1,31_ = 87.43, *P <* 0.000001) compared to baseline values, indicating effective retention of the conditioned fear response. Neither drug nor lesion affected duration or frequency of freezing (Figure 8(b)). No differences were observed among groups in number of defecations. As for USVs, the experimental groups just tended to differ (H = 6.71, *P* = 0.08 by Kruskal-Wallis test), with Don-Sap and Sal-Sap lesioned rats emitting fewer USVs than sham rats. Further USV parameters are reported in Additional file 4.

#### Tone test

All rats showed a significant increase in duration (F_1,31_ = 113.44, *P <* 0.000001) and frequency (F_1,31_ = 52.61, *P <* 0.000001) of freezing during tone reexposure in comparison to baseline values, indicating once again an effective retention of the conditioned fear response. Neither drug nor lesion affected duration or frequency of freezing (Figure 8(c)). No differences were observed among groups in number of defecations. USVs were scarcely emitted by all rats, regardless of drug and lesion conditions (H = 2.53, *P* = 0.47 by Kruskal-Wallis test) (Figure 8(c)). Further USV parameters are reported in Additional file 5.

## Discussion

As ACh reduction is a morphofunctional hallmark in AD, AChE-Is, such as donepezil, are used to improve AD-related cognitive deterioration [7]. Besides symptomatic effects, many *in vitro* studies have shown that AChE-Is might exert neuroprotective action against neurotoxic agents, such as glutamate and Aβ plaques [8-11,15-18]. Unfortunately, only a few *in vivo* experiments have confirmed AChE-I pre-lesion neuroprotective action [12-14,26,27]. Interestingly, the present study demonstrates a marked neuroprotective effect of donepezil against neurodegeneration and cognitive impairment induced by cholinergic depletion in rats. Donepezil pretreatment did not induce any biochemical or behavioral modification in sham-lesioned rats, which is in accord with the findings of Saxena *et al*. [14] and Su *et al*. [27]. We previously demonstrated that healthy rats treated with donepezil *during* behavioral testing showed enhanced memory and explorative functions in RAM and OF, as well as reduced anxiety levels in EPM, but they did not exhibit any improvement in spatial span, motivational levels and associative learning [41]. Thus, although donepezil treatment *during* testing is able to improve cognitive performance even in unlesioned animals, donepezil treatment *before* testing (pre-treatment) exerts neuroprotective action only in the presence of lesion or insult.

Cholinergic depletion *per se* (Sal-Sap group) increased hippocampal and neocortical caspase-3 activity and impaired working memory performance in RAM and spatial change detection in OF, altered social discrimination performance in PSNT and reduced USV production during FC, but it did not affect anxiety levels in EPM and FC acquisition. The behavioral results in our study are in line with those reported in previous studies of memory deficits [20,29,42,43] and impaired production of USVs [38,40,43] and no effects on anxiety levels following 192 IgG-Sap lesion [20,44]. Our present is the first one that explains caspase-3 activity in the target areas of cholinergic projections. Our results demonstrate that donepezil pretreatment (Don-Sap group) was able to counteract neurodegeneration associated with cholinergic depletion. In fact, hippocampal and neocortical caspase-3 levels observed in Don-Sap rats were significantly decreased in comparison to Sal-Sap. This result is in line with previous studies using donepezil pre-treatment in different experimental paradigms. In a glutamate excitotoxicity study, donepezil pre-treatment significantly prevented caspase-3 activation, contributing to internalization and downregulation of *N*-methyl-D-aspartate (NMDA) receptors through α_7_ nAChR stimulation [15]. Moreover, following STZ lesioning, donepezil pre-treatment significantly prevented hippocampal and neocortical caspase-3 activity [14]. The reduction of hippocampal and neocortical caspase-3 activity by donepezil pre-treatment following cholinergic depletion adds further evidence to donepezil neuroprotective action. This effect could be ascribed to the up-regulation of nAChRs and to the consequent activation of the anti-apoptotic nAChR/PI3K/Akt/Bcl2 pathway, as previously hypothesized [8,17]. Namely, several studies have demonstrated the involvement of nAChRs in donepezil-induced neuroprotection. In fact, in contrast to the AChE-Is galantamine and tacrine, prolonged donepezil administration induces not only a functional up-regulation but also an increased expression of nAChRs [9,17]. Importantly, nAChR up-regulation by donepezil is able to activate intracellular anti-apoptotic secondary messenger systems, such as the nAChR/PI3K pathway, that protect neurons against neuron death [17]. Furthermore, the neuroprotective effects of donepezil appear to be mediated by σ_1_ receptor activation that result in intracellular modulation of Ca^2+^ mobilization and activation of phospholipase C (PLC)/protein kinase C (PKC) transduction pathways, attenuating the effects of mitochondrial or endoplasmic reticulum dysfunction [12,13].

The neuroprotective action of donepezil pre-treatment on hippocampal and neocortical neurodegeneration could account for the improved RAM and OF spatial performance of the pre-treated lesioned animals. Namely, in RAM, Don-Sap rats exhibited a lower number of errors and a higher spatial span than Sal-Sap rats, reaching the mnesic performance levels of sham-lesioned rats.

Beneficial effects of donepezil treatment on spatial working memory have been demonstrated in many experimental models. In fact, donepezil administration reduced RAM errors in aged or scopolamine-treated animals [45,46]. Working memory improvement has been reported in aged mice treated with donepezil or a nicotinic agonist α_7_ (starting one week before behavioral training) [23]. In addition, in a rat model of cholinergic depletion, donepezil administration (starting 1 week before and lasting 9 weeks after lesioning) mitigated procedural and working memory deficits in RAM [20]. In hypoxia and Aβ toxicity models, donepezil pre-treatment prevented spatial working memory deficits [12,13].

Even in OF, although cholinergic depletion *per se* (Sal-Sap group) significantly affected spatial discrimination abilities, donepezil pre-treatment (Don-Sap group) prevented the spatial memory deficits. Consistent with the previously described impaired spatial change and preserved novelty recognition following Sap lesions [20,42,43,47], all lesioned rats detected the presence of the novel object, just as sham-lesioned rats did. In fact, though spatial change detection implies efficient spatial mapping, novelty detection implies recognition of the salient object, thus minimizing the effect of the spatial and contextual factors [38,48,49]. Interestingly, the preserved abilities in detecting spatial change of Don-Sap rats reveal the specific neuroprotective effect of donepezil on a subtle spatial deficit induced by the cholinergic lesion and are in agreement with donepezil neuroprotective properties on spatial mnesic performances in different brain damage models [12-14,26,27].

In all animals, the shock exposure induced a clear freezing response during FC training, and the conditioned freezing response was retained during context and tone tests. Although the lesion did not alter freezing response, it decreased the concomitant production of 22-kHz USVs. Interestingly, donepezil pre-treatment during FC training just tended to rescue the defect of fear-associated USVs, which were nearly totally suppressed in Sal-Sap rats. The 22-kHz USVs are part of the rodent defensive repertoire and are emitted in response to negative experiences, such as foot-shock, presence of predators or startling noise exposure [30-32]. These USVs are closely associated with freezing response to actual or potential threats and might serve as “alarm calls” for conspecificity. There is converging evidence that Sap lesions selectively impair the functionality of neural systems regulating USVs [38,40,43]. Overall, our data are in agreement with the previously described disassociation between intact freezing response and impaired USV production in cholinergically depleted rats [38]. Intriguingly, donepezil pre-treatment (Don-Sap group) appeared to mitigate the USV suppression of lesioned rats during FC training. Thus, our data corroborate the added value of USV measurement, which provides additional information about the emotional state of the animals in situations of coping with inescapable aversive stimuli.

Also, social interactions and recognition of conspecificity are fundamental and adaptive components of the behavioral repertoire of numerous species. Similarly to social transmission of food preference, social recognition memory, which relies on the functional activation of hippocampal and prelimbic cortical circuitry [50], is impaired in the early phases of AD. Clinical trials have demonstrated that donepezil enhances AD patients’ social interaction, engagement and interest [51]. On these bases, in the present research, we investigated for the first time donepezil pre-treatment effects on social skills using the three-chamber test. During sociability tests, all animals showed a clear social motivation. In fact, all of them preferred the conspecific stranger, as they spent more time and traveled longer distances in the chamber containing the social stimulus. These results fit with those obtained in the sociability test described by Riedel *et al*. [50], but they seem to be in disagreement with those obtained by Savage *et al*. [47]. On the basis of use of a social interaction test, these authors reported a significant reduction in active interactions following selective cholinergic depletion of the neocortex in rats. Notably, at variance with the social interaction test, sociability does not allow any direct contact with the social stranger. Thus, in the present study, the indirect social interactions and the scarce salience of the empty compartment featuring sociability appear to have facilitated the exploration of the stranger by the lesioned rats.

During PSNT, though sham rats showed a clear preference for the novel stranger, the cholinergically depleted animals did not exhibit overt social novelty. This result has to be interpreted as a specific social recognition memory deficit (as described by Riedel *et al*. [50]), because the OF lesioned rats did recognize the novel object, which is in line with the findings reported by Savage *et al*. [47]. Although Riedel *et al*. [50] reported that donepezil administration succeeded in rescuing social memory scopolamine-induced deficits, donepezil pre-treatment failed to prevent PSNT deficits in lesioned rats in our present study. The different results could be explained by methodological differences, such as treatment time (acute *vs.* pre-treatment), behavioral protocols (short-term *vs.* long-term memory) and cholinergic manipulations (scopolamine *vs.* Sap). Notably, hippocampal and neocortical cholinergic deafferentation by Sap results in massive dysregulation of other neurotransmitter systems, such as dopaminergic and glutamatergic ones, which is known to contribute to social discrimination [46,52,53].

Our current research reveals that donepezil pre-treatment is able to reduce hippocampal and neocortical caspase-3 activity, thus preventing neuron degeneration, and to exert beneficial effects on specific behavioral deficits induced by cholinergic depletion. As indicated in previous studies, donepezil neuroprotective effects might be mediated by many protective mechanisms, such as nAChR upregulation and activation of the nAChR/PI3K pathway [8,15,17] and the σ_1_ receptor/PLC/PKC pathway [12,13]. Such effects result in a reduction of neurotoxicity linked to NMDA receptor–mediated Ca^2+^ influx, oxidative stress and caspase-3 activity [14,15]. Furthermore, donepezil exerts a protective action against Aβ toxicity [7]. In fact, in AD patients, Aβ plaques colocalize with nAChRs and α_4_ and α_7_ nAChR expression is reduced. In CA1, the α4-nAChR activation causes γ-aminobutyric acid (GABA) release from interneurons that inhibit pyramidal neurons, whereas the activation of α_7_ nAChRs in pyramidal neurons results in Ca^2+^ influx, presynaptic neurotransmitter release and postsynaptic depolarization [7]. Thus, Aβ interferes with nAChR activity, and the consequent increase of glutamate and decrease of GABA induce glutamate excitotoxicity and excitation–inhibition imbalance, altering the fine-tuning of hippocampal firing [6]. In AD patients, increase of caspase-3 activity has been reported in the hippocampal and neocortical postsynaptic density fractions [54]. Also, in the Tg2576 AD mouse model, the increase of caspase-3 in hippocampal postsynaptic compartment leads to alteration of synaptic plasticity and dendritic spine loss. In parallel, the Sap-induced cholinergic depletion mimicking Aβ interference might cause an alteration of the excitation–inhibition balance and produce excitotoxic damage in hippocampal and neocortical neurons, which accumulate active caspase-3. On these bases, the question is: How can donepezil neuroprotection be exerted? By inhibiting AChE activity, the donepezil pre-treatment might reduce the GABAergic alterations and prevent glutamatergic excitotoxicity and the excitation–inhibition imbalance. In this way, caspase-3 accumulation would be decreased, contributing to the maintenance of hippocampal and neocortical functioning.

## Conclusions

The present results show for the first time that donepezil pre-treatment is able to slow down the memory deficits induced by cholinergic depletion and to reduce caspase-3 accumulation in hippocampal and neocortical areas. Although further studies deepening understanding of the molecular mechanisms of donepezil neuroprotective action are needed, the present results are promising and may lead to the development of novel strategies for prevention and therapy of neurodegenerative diseases.

## Abbreviations

ACh: Acetylcholine; AChE-I: Acetylcholinesterase inhibitor; AD: Alzheimer’s disease; ANOVA: Analysis of variance; AP: Anteroposterior; Aβ: Amyloid β; ChAT: Choline acetyltransferase; Don-Sap: Donepezil-treated immunoglobulin G saporin-lesioned rats; Don-Sham: Donepezil-treated sham-lesioned rats; DV: Dorsoventral; EPM: Elevated plus maze; FC: Fear conditioning; MCI: Mild cognitive impairment; ML: Mediolateral; MS: Medial septum; nAChR: Nicotinic acetylcholine receptor; NBM: Nucleus basalis magnocellularis; OF: Open field with objects; PBS: Phosphate-buffered saline; PI3K: Phosphatidylinositol 3-kinase; PKC: Protein kinase C; PLC: Phospholipase C; PSNT: Preference for social novelty test; RAM: Radial arm maze; Sal-Sap: Saline-treated immunoglobulin G saporin-lesioned rats; Sal-Sham: Saline-treated sham-lesioned rats; Sap: 192 immunoglobulin G saporin; STZ: Streptozotocin; USV: Ultrasonic vocalization.

## Competing interests

The authors have no conflicts of interest to declare.

## Authors’ contributions

DC, LP and MDA made substantial contributions to the conception and design of the study. PDB performed immunotoxic lesioning and histological analyses. DC, PC and AMT treated the animals and performed behavioral evaluations. DO contributed to acquisition and analysis of USV data. AN performed biochemical analyses. DC and AN undertook the statistical analyses. DC, LP, MDA and FDA were involved in drafting the manuscript and revising it critically for important intellectual content. All authors contributed to the interpretation of data and gave their final approval of the manuscript version to be published.

## Supplementary Material

Additional file 1 Figure representing sham rats’ data. Click here for file

Additional file 2 Table of EPM data. Click here for file

Additional file 3 Table of the main spectrographic parameters of USVs emitted during FC training. Click here for file

Additional file 4 Table of the main spectrographic parameters of USVs emitted during the context test. Click here for file

Additional file 5 Table of the main spectrographic parameters of USVs emitted during tone test. Click here for file
