# Supplementary material for: Neuroprotective effects of donepezil against cholinergic depletion
Source: Alzheimers Res Ther. 2013 Oct 24;5(5):50. doi: 10.1186/alzrt215 (PMC3978431; doi:10.1186/alzrt215)
Supplement: Additional file 1 — Figure representing sham rats’ data. [file alzrt215-S1.pdf]

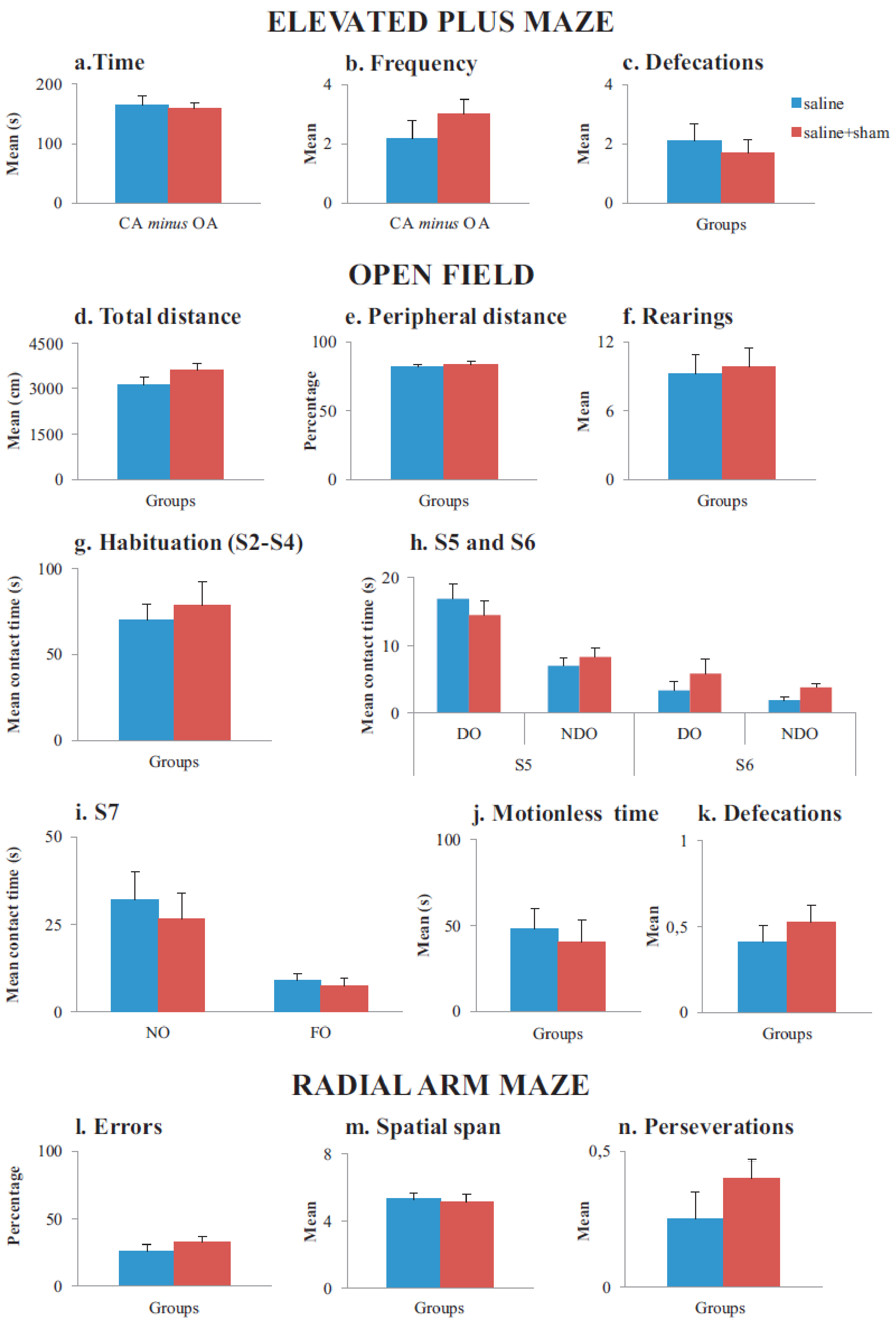
ELEVATED PLUS MAZE
a.Time
Mean (s)
200
100
0
CA minus OA
b. Frequency
Mean
4
2
0
CA minus OA
c. Defecations
Mean
4
2
0
Groups
saline
saline+sham
OPEN FIELD
d. Total distance
Mean (cm)
4500
3000
1500
0
Groups
e. Peripheral distance
Percentage
100
50
0
Groups
f. Rearings
Mean
12
6
0
Groups
g. Habituation (S2-S4)
Mean contact time (s)
100
50
0
Groups
h. S5 and S6
Mean contact time (s)
20
10
0
DO
NDO
DO
NDO
S5
S6
i. S7
Mean contact time (s)
50
25
0
NO
FO
j. Motionless time
Mean (s)
100
50
0
Groups
k. Defecations
Mean
1
0,5
0
Groups
RADIAL ARM MAZE
l. Errors
Percentage
100
50
0
Groups
m. Spatial span
Mean
8
4
0
Groups
n. Perseverations
Mean
0,5
0
Groups

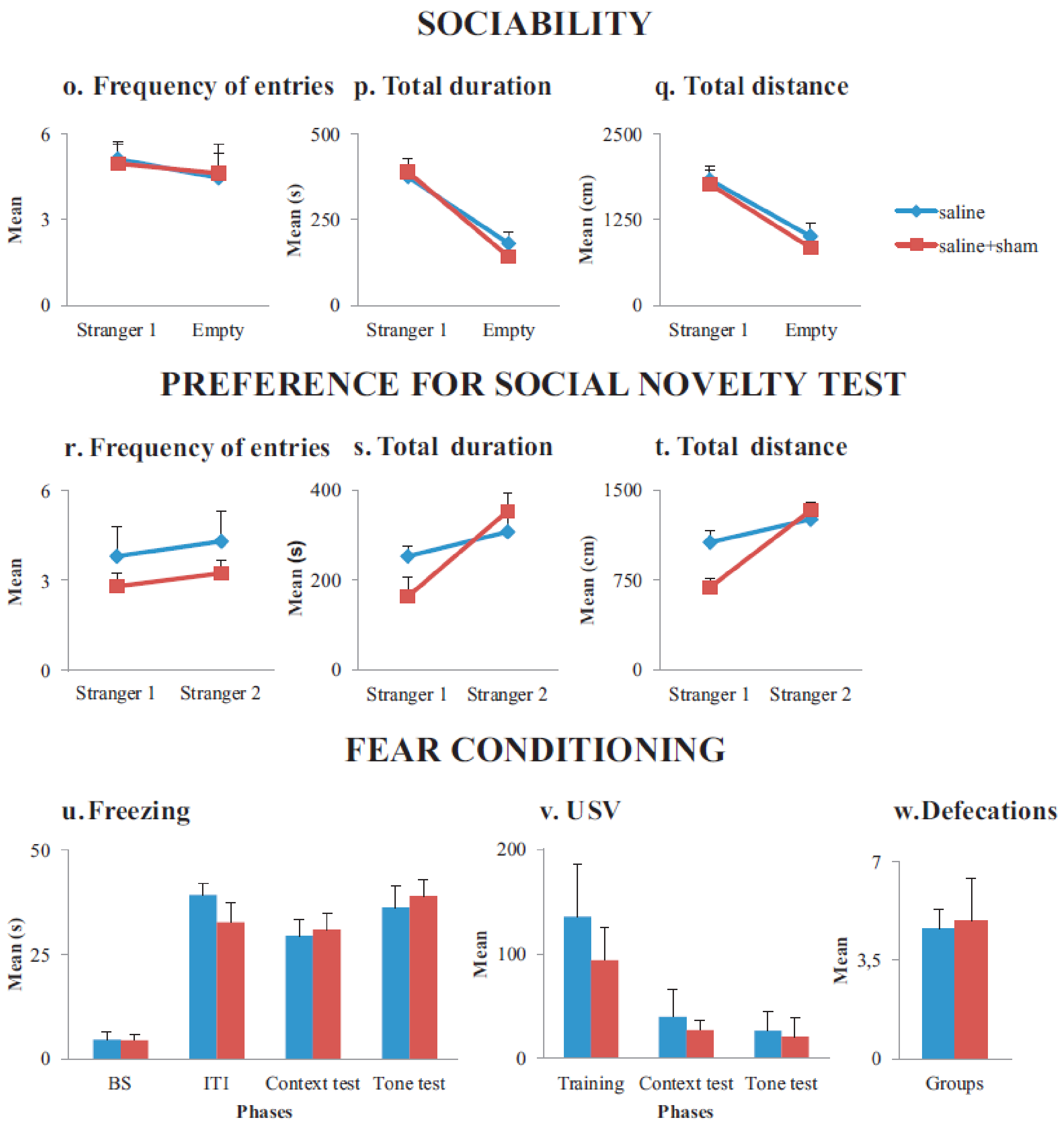

Additional file 1. Data of intact rats treated with saline alone (saline) and rats treated with saline and subjected to sham lesion (saline+sham) are depicted. ELEVATED PLUS MAZE parameters: mean time (a) and frequency of entries (b) in the Closed Arms (CA) *minus* Open Arms (OA); number of defecations (c). **OPEN FIELD parameters:** total (d) and peripheral (e) distances travelled in session 1 (S1); number of rearings (f); mean contact time with objects during habituation (S2-S4) (g), spatial change (S5, S6) (h), and novelty (S7) (i); motionless time (j); number of defecations (k). **RADIAL ARM MAZE parameters:** percentage of errors (l); spatial span (m); perseverations (n). **SOCIABILITY parameters:** frequency of entries (o); duration of time spent (p) and distance travelled in each chamber (q). **PREFERENCE FOR SOCIAL NOVELTY TEST parameters:** frequency of entries (r); duration of time spent (s) and distance travelled in each chamber (t). **FEAR CONDITIONING parameters:** freezing time (u); number of ultrasonic vocalizations, USV (v); defecations (w). Not statistically significant differences were found between saline and saline+sham rats. Data are expressed as mean ± *SEM.*
