# Supplementary material for: Neuroprotective effects of donepezil against cholinergic depletion
Source: Alzheimers Res Ther. 2013 Oct 24;5(5):50. doi: 10.1186/alzrt215 (PMC3978431; doi:10.1186/alzrt215)
Supplement: Additional file 2 — Table of EPM data. [file alzrt215-S2.pdf]

**EPM parameters**	**Time**	
	**Open Arms**	**Closed Arms**
Don-Sham	20.43 ± 11.05	223.86 ± 19.38
Sal-Sham	16.00 ± 4.64	178.08 ± 11.28
Don-Sap	16.88 ± 6.63	180.63 ± 17.06
Sal-Sap	44.63 ± 12.65	186.00 ± 21.13
	**Frequency**	
	**Open Arms**	**Closed Arms**
Don-Sham	1.57 ± 0.25	3.43 ± 0.69
Sal-Sham	2.00 ± 0.35	4.58 ± 0.57
Don-Sap	1.75 ± 0.31	5.43 ± 0.49
Sal-Sap	2.63 ± 0.50	4.25 ± 0.88
	**Total defecations**	
Don-Sham	3.45 ± 0.92	
Sal-Sham	1.83 ± 0.65	
Don-Sap	2.67 ± 0.43	
Sal-Sap	2.00 ± 0.69	

Additional file 2. Table of EPM data: frequency of entries and total time spent in the open and closed arms; number of defecations. Values represent mean ± *SEM*.
