# Supplementary material for: Neuroprotective effects of donepezil against cholinergic depletion
Source: Alzheimers Res Ther. 2013 Oct 24;5(5):50. doi: 10.1186/alzrt215 (PMC3978431; doi:10.1186/alzrt215)
Supplement: Additional file 3 — Table of the main spectrographic parameters of USVs emitted during FC training. [file alzrt215-S3.pdf]

SBJs EMITTING	Baseline	ITI	Tone+Shock
Don-Sham	0/7	4/7	4/7
Sal-Sham	1/12	9/12	9/12
Don-Sap	0/8	4/8	4/8
Sal-Sap	0/8	1/8	0/8
**DURATION (ms)**			
Don-Sham	-	1.05 ± 0.08	1.13 ± 0.11
Sal-Sham	0.30	1.15 ± 0.15	1.09 ± 0.12
Don-Sap	-	1.23 ± 0.13	1.23 ± 0.17
Sal-Sap	-	1.82	-
**FREQUENCY MODULATION (kHz)**			
Don-Sham	-	5.10 ± 0.86	4.88 ± 1.08
Sal-Sham	6.96	5.36 ± 0.81	5.12 ± 0.75
Don-Sap	-	5.10 ± 0.86	4.88 ± 1.08
Sal-Sap	-	7.32	-
**PEAK FREQUENCY (kHz)**			
Don-Sham	-	23.95 ± 0.75	22.91 ± 0.80
Sal-Sham	22.70	23.35 ± 0.78	23.72 ± 0.47
Don-Sap	-	25.41 ± 1.13	25.24 ± 1.28
Sal-Sap	-	24.50	-
**PEAK AMPLITUDE (dB)**			
Don-Sham	-	-34.56 ± 5.83	-35.06 ± 6.14
Sal-Sham	-55.56	-34.21 ± 3.95	-34.76 ± 3.11
Don-Sap	-	-39.56 ± 3.39	-38.27 ± 3.17
Sal-Sap	-	-33.68	-

Additional file 3. Table of the main spectrographic parameters of USVs emitted during FC training. Values represent mean ± *SEM*.
