# Supplementary material for: Neuroprotective effects of donepezil against cholinergic depletion
Source: Alzheimers Res Ther. 2013 Oct 24;5(5):50. doi: 10.1186/alzrt215 (PMC3978431; doi:10.1186/alzrt215)
Supplement: Additional file 4 — Table of the main spectrographic parameters of USVs emitted during the context test. [file alzrt215-S4.pdf]

**SBJs EMITTING**	**CONTEXT**
Don-Sham	3/7
Sal-Sham	7/12
Don-Sap	2/8
Sal-Sap	0/8
**DURATION (ms)**	
Don-Sham	1.21 ± 0.14
Sal-Sham	1.19 ± 0.19
Don-Sap	1.50 ± 0.71
Sal-Sap	-
**FREQUENCY MODULATION (kHz)**	
Don-Sham	4.99 ± 1.09
Sal-Sham	4.38 ± 3.28
Don-Sap	2.75 ± 4.48
Sal-Sap	-
**PEAK FREQUENCY (kHz)**	
Don-Sham	23.81 ± 0.80
Sal-Sham	23.41 ± 0.43
Don-Sap	26.67 ± 2.82
Sal-Sap	-
**PEAK AMPLITUDE (dB)**	
Don-Sham	-51.48 ± 4.71
Sal-Sham	-36.82 ± 3.24
Don-Sap	-43.29 ± 7.73
Sal-Sap	-

Additional file 4. Table of the main spectrographic parameters of USVs emitted during the context test. Values represent mean ± *SEM*.
