# Supplementary material for: Neuroprotective effects of donepezil against cholinergic depletion
Source: Alzheimers Res Ther. 2013 Oct 24;5(5):50. doi: 10.1186/alzrt215 (PMC3978431; doi:10.1186/alzrt215)
Supplement: Additional file 5 — Table of the main spectrographic parameters of USVs emitted during tone test. [file alzrt215-S5.pdf]

SBJs EMITTING	No tone	Tone exposure
Don-Sham	0/7	4/7
Sal-Sham	3/12	4/12
Don-Sap	1/8	1/8
Sal-Sap	1/8	1/8
**DURATION (ms)**		
Don-Sham	-	1.29 ± 0.63
Sal-Sham	0.86 ± 0.29	1.19 ± 0.66
Don-Sap	0.96	2.05
Sal-Sap	1.66	1.66
**FREQUENCY MODULATION (kHz)**		
Don-Sham	-	4.60 ± 1.48
Sal-Sham	4.20 ± 1.37	4.70 ± 1.59
Don-Sap	2.60	3.68
Sal-Sap	2.53	2.25
**PEAK FREQUENCY (kHz)**		
Don-Sham	-	22.38 ± 1.22
Sal-Sham	23.45 ± 0.65	22.58 ± 0.41
Don-Sap	23.57	23.27
Sal-Sap	22.33	22.47
**PEAK AMPLITUDE (dB)**		
Don Sham	-	-45.96 ± 5.31
Sal Sham	-45.29 ± 6.27	-40.52 ± 4.54
Don Sap	-56.88	-47.01
Sal Sap	-39.62	-34.04

Additional file 5. Table of the main spectrographic parameters of USVs emitted during tone test. Values represent mean ± *SEM*.
